# Taurine Transporter Gene Expression in Mononuclear Blood Cells of Type 1 Diabetes Patients

**DOI:** 10.1155/2016/7313162

**Published:** 2016-02-03

**Authors:** Zaleida Napoli, Giuseppe Seghieri, Loria Bianchi, Roberto Anichini, Alessandra De Bellis, Ilaria Campesi, Ciriaco Carru, Stefano Occhioni, Angelo Zinellu, Flavia Franconi

**Affiliations:** ^1^Department of Clinical Chemistry, S. Jacopo Hospital, 51100 Pistoia, Italy; ^2^Agenzia Regionale Sanità, Toscana, Florence, Italy; ^3^Accademia Medica Pistoiese F. Pacini, 51100 Pistoia, Italy; ^4^Diabetes Unit, S. Jacopo Hospital, 51100 Pistoia, Italy; ^5^Department of Biomedical Sciences, University of Sassari, 07100 Sassari, Italy

## Abstract

*Background*. Taurine transporter gene expression (RNA-TauT) has a role in retinal cell function and is modulated* in vitro* and* in vivo* by hyperglycemia and/or oxidative stress. This study was aimed at testing whether RNA-TauT gene expression is modified in blood mononuclear peripheral cells (MPCs) of type 1 diabetic patients, is related to plasma markers of oxidative stress or endothelial dysfunction, or, finally, is related to presence of retinopathy.* Methods*. RNA-TauT was measured in MPCs by real-time PCR-analysis in 35 type 1 diabetic patients and in 33 age- and sex-matched controls, additionally measuring plasma and cell taurine and markers of oxidative stress and endothelial dysfunction.* Results*. RNA-TauT, expressed as 2^−ΔΔCt^, was significantly higher in MPCs of type 1 diabetic patients than in controls [median (interquartile range): 1.32(0.31) versus 1.00(0.15); *P* = 0.01]. In diabetic patients RNA-TauT was related to HbA1c (*r* = 0.42; *P* = 0.01) and inversely to plasma homocysteine (*r* = −0.39; *P* = 0.02) being additionally significantly higher in MPCs of patients without retinopathy [(*n* = 22); 1.36(0.34)] compared to those with retinopathy [(*n* = 13); 1.16(0.20)], independently from HbA1c or diabetes duration.* Conclusions*. RNA-TauT gene expression is significantly upregulated in MPCs of type 1 diabetes patients and is related to HbA1c levels and inversely to plasma homocysteine. Finally, in diabetes patients, RNA-TauT upregulation seems to be blunted in patients with retinopathy independently of their metabolic control or longer diabetes duration.

## 1. Introduction

The sulfur amino acid taurine is the most abundant free amino acid in the human body and is involved in many fundamental biological functions such as antioxidation, osmoregulation, and Ca++ transport regulation. It also has anti-inflammatory effects [1, 2]. In humans, the main source of taurine is the diet, the rate of endogenous synthesis being relatively low.

Taurine has preventive effects against the incidence of diabetes, improving insulin sensitivity and secretion in animal models and in humans [3–5]. In addition, taurine plays an important role in retinal function, at least in animal models, as demonstrated by the dated observation about a close relationship between taurine deficiency and retinal degeneration in the cat [6]. This relationship assumes an ever greater significance in diabetes since dietary taurine supplementation prevents glial alterations in the retina of diabetic rats [7] and, consequently, ameliorates diabetic microangiopathy, including retinopathy [8–10].

Taurine is 10^3^-fold more concentrated in the intracellular space, due to the action of a specific sodium-dependent transporter (TauT). The latter is upregulated by several conditions, including hypertonicity, low extracellular taurine, and oxidative stress [11, 12], all of which are frequently found in diabetic patients [13–15]. In regard to hypertonicity, it is known that taurine is an osmolyte involved in cell volume regulation [2, 4] and hyperosmolality associated with diabetic hyperglycemia could trigger increased gene expression of TauT. It has moreover recently been shown that blood mononuclear peripheral cells (MPCs) obtained from type 2 diabetic patients overexpress the TauT gene, and this overexpression seems to be blunted in the presence of retinopathy [16].

The aim of this study was to test whether TauT mRNA gene expression is also modified in MPCs of type 1 diabetic patients, who are, differently from type 2 diabetic patients, characterized by a younger age, longer duration of disease, and requirement of insulin therapy since the onset of disease. Further objectives of this study were investigating whether TauT mRNA expression is modulated by plasma levels of oxidative stress or endothelial dysfunction markers or, finally, whether it is related to the presence of retinopathy.

## 2. Materials and Methods

### 2.1. Patients and Controls

Type 1 diabetic patients were recruited from among the diabetic subjects who consecutively attend the Diabetes Outpatient Clinic of the Pistoia Hospital. Retinopathy was ascertained by retinal fluorescein angiography as previously described [17].

Renal pathology was investigated by estimation of urinary albumin to creatinine ratio using spot urine samples, and all patients were in the normoalbuminuric range except for one.

Macroangiopathy, ascertained by patients' previous medical history and clinical-instrumental criteria, was excluded in all cases but two. Clinical evidence of peripheral neuropathy was excluded in all cases.

Age- and sex-matched controls were recruited from among hospital personnel or their relatives. Neither patients nor controls took extra vitamins or drugs potentially known to modify both taurine and its cell transporter.

This study was approved by the Ethics Committee of the Pistoia Hospital and all subjects gave their written informed consent prior to their inclusion in the study.

### 2.2. Biochemical Assays

We measured fasting plasma glucose and glycated hemoglobin (HbA1c) (resp., by the glucose oxidase method and by HPLC, Bio-Rad Laboratories, USA) in both patients and controls. Plasma levels of malonyldialdehyde (MDA) were measured spectrophotometrically by the thiobarbituric method as previously described [18]. Carbonylated proteins were quantified spectrophotometrically after protein precipitation with 10% TCA as described by Fagan et al. [19]. Plasma levels of asymmetric dimethylarginine (ADMA), symmetric dimethylarginine (SDMA), and arginine were measured by field-amplified sample injection capillary electrophoresis as previously reported [20] and plasma homocysteine by fluorescence polarization immunoassay (FPIA, Abbott, Italy). Serum and cell taurine, expressed as nmol/10^6^ cells, were measured by capillary electrophoresis with laser-induced fluorescence detection [21, 22]. Each sample was assayed in duplicate.

### 2.3. RNA-Taurine Transporter Gene Expression

Blood mononuclear peripheral cells (MPCs, lymphocytes and monocytes) were extracted from 9 mL of whole blood in each subject by Accuspin System-Histopaque-1077 (Sigma-Aldrich, Italy) and separated into two aliquots: one aliquot was resuspended in 200 *μ*L of RNA*later* RNA stabilization Reagent (Qiagen, UK) for RNA extraction, and one aliquot was resuspended in 200 *μ*L of PBS solution for intracellular content determinations. Each sample contained a median count of 2.59*∗*10^6^ [interquartile range: 0.8*∗*10^6^] cells/mL, comprised of 2.09*∗*10^6^ [interquartile range: 0.7*∗*10^6^] lymphocytes/mL and 0.5*∗*10^6^ [interquartile range: 0.12*∗*10^6^] monocytes/mL. The average amount of total cells obtained from extraction in each aliquot was 4.1*∗*10^6^ (19.4*∗*10^3^ cells/*μ*L, interquartile range: 18.3*∗*10^3^ cells/*μ*L), with an average cellular extraction yield of 43.37%  ±  6.68% (95% IC; from a minimum of 0.55% to a maximum of 96.4%). TauT mRNA was quantified by real-time (RT) PCR (Rotorgene 6000, Qiagen, UK), as previously described [16], using hypoxanthine-guanine phosphoribosyltransferase (HPRT) as house-keeping gene. Primers' sequence for TauT (SLC6A6; gene accession number: NM_001134367) was 5′-TATCTGTATCCTGACATCACCCG-3′ (forward) and 5′-CCCAGGCAGATGGCATAAGAG-3′ (reverse), while for HPRT it (gene accession number: NM_000194) was 5′-GAAGGTGAAGGTCGGAGT-3′ (forward) and 5′-GAAGATGGTGATGGGATTTA-3′ (reverse) [23].

Relative quantification was performed using the comparative cycle threshold (Ct) method after determining the Ct values for the reference (HPRT) and target gene (TauT) in each sample, according to the 2^−ΔΔCt^ method [24]. To assess whether two amplifications have the same efficiency (required condition for 2^−ΔΔCt^ method), ten standard curves were generated by reverse transcription of Human Reference Total RNA (Agilent Technologies, USA) and amplification of scalar 1 : 5 dilutions of cDNA. Standard curves showed efficiencies between 0.79 ± 0.02 (IC 95%, CV = 4.15%) for HPRT and 0.76 ± 0.03 (IC 95%, CV = 5.36%) for TauT, with a linearity range between 100 and 0.16 *μ*g/mL of total RNA.

### 2.4. Statistical Methods

Comparisons among groups, often including nonnormally distributed elements, were analyzed using the Wilcoxon-Mann-Whitney test and relations among variables were tested by Spearman's correlation coefficients. Nonnormally distributed variables were expressed as median [interquartile ranges]. The significance of *P* value was set at <0.05. All statistical analyses were carried out by means of SAS software for Windows, version 9.3 (SAS Institute Inc., Cary, NC, USA).

## 3. Results 

**Figure 1:**
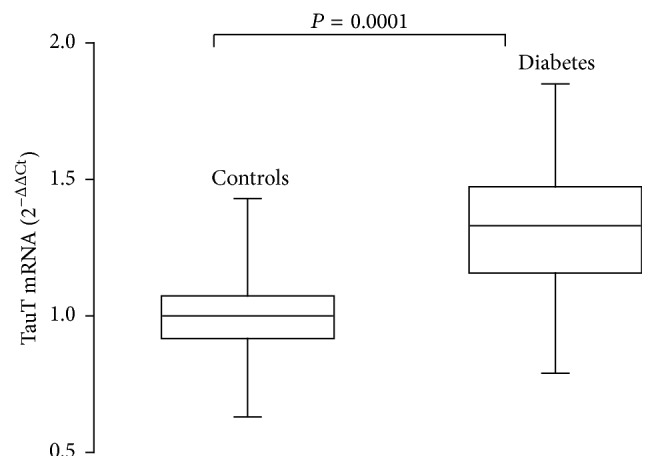
Box and whisker plots of TauT mRNA gene expression in mononuclear peripheral blood cells of type 1 diabetes patients and of controls.

Controls and diabetic patients were matched for age, sex, and body mass index (BMI). Fasting plasma glucose and HbA1c were significantly higher in diabetic patients. The TauT mRNA gene expression in MPCs was significantly higher, by about 32%, in diabetic patients, compared to healthy volunteers (Table 1, Figure 1).

**Figure 2:**
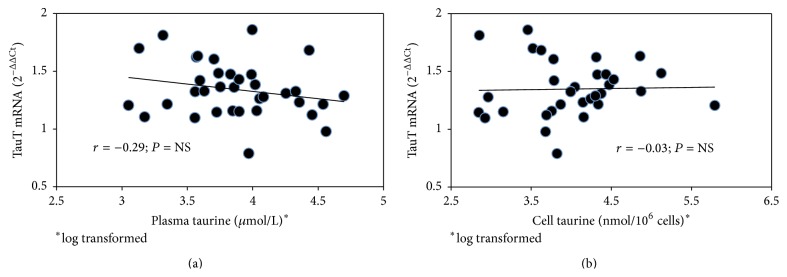
Relation between TauT mRNA gene expression and plasma (a), or cell taurine (b), in MPCs from type 1 diabetic patients.

**Figure 3:**
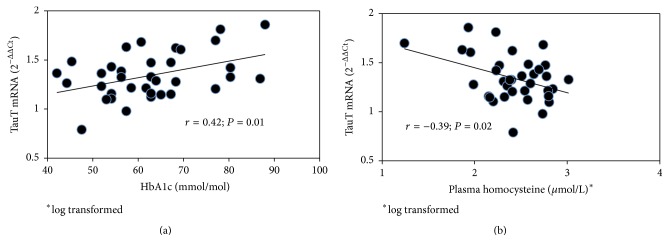
Relation between TauT mRNA gene expression and HbA1c (a), or plasma homocysteine (b), in MPCs from type 1 diabetic patients.

Among controls no correlation was observed between TauT mRNA gene expression and HbA1c, fasting plasma glucose, homocysteine, malonyldialdehyde, carbonyl groups, ADMA, and SDMA (Table 2). TauT mRNA was not significantly related to plasma- or intracellular taurine (Table 2, Figure 2). In diabetic patients, the TauT mRNA gene was positively related to HbA1c (*r* = 0.42; *P* = 0.01, Figure 3(a)), whereas it was not related to fasting plasma glucose (*r* = 0.22; *P* = NS, Table 2). Duration of diabetes was inversely related to TauT mRNA gene expression, even if not significantly (*r* = −0.26; *P* = 0.06). Finally, TauT mRNA gene expression was inversely related to plasma homocysteine (*r* = −0.39; *P* = 0.02); (Table 2, Figure 3(b)).

All diabetic patients were on insulin therapy and plasma insulin levels, evaluated together with TauT mRNA, were not significantly related to each other (*r* = −0.058; *P* = NS).

**Figure 4:**
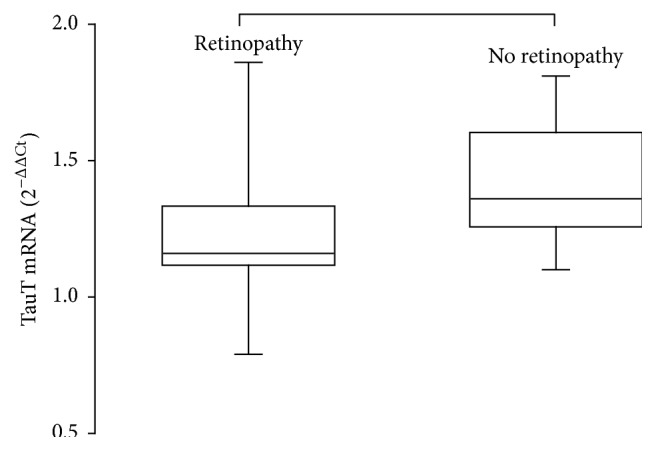
Box and whisker plots of TauT mRNA gene expression in mononuclear peripheral blood cells of type 1 diabetes with or without retinopathy.

Patients with retinopathy had a longer duration of disease and a significantly lower TauT mRNA gene expression than the subjects without retinopathy, while no significant difference was observed for any other studied plasma marker between these two groups (Table 3, Figure 4). In addition, higher TauT mRNA gene expression remained significantly related to absence of retinopathy, after adjusting for HbA1c and disease duration by a multiple regression analysis (*P* = 0.01).

Finally, no significant difference was found between plasma or cell taurine concentration of retinopathic versus nonretinopathic patients, although the intracellular level tended to be higher in the latter (Table 3). Again, in the whole group of diabetes patients, after a multivariate model with HbA1c and length of disease as covariates, presence of retinopathy was independently and significantly associated with a decrease in TauT mRNA (*P* = 0.02).

## 4. Discussion 

This study provides evidence that type 1 diabetes patients have a significant increase (by about 30%) in TauT mRNA gene expression in MPCs, when compared with age- and sex-matched control healthy subjects, and that such expression is directly related to HbA1c, suggesting the hypothesis that diabetes,* “in vivo,”* may be able to chronically induce cell TauT mRNA gene expression, hypothetically as a defense measure, to improve cell homeostasis against the exposure to chronically higher glucose levels. These data observed* “in vivo”* seem to be quite different from the findings obtained in experimental models* “in vitro”* where acute exposure to hyperglycemia and oxidative stress downregulate TauT mRNA gene expression in some cellular types [25–28].

In addition, a possible action of prevailing insulin levels on TauT cell gene expression was excluded by the absence of any relationship between plasma insulin concentrations and TauT gene expression in diabetic patients.

At variance with previous observations [29], no significant differences were observed between plasma or cell taurine concentrations from type 1 diabetic patients compared to control subjects. It is well known, however, that plasma levels of taurine are highly dependent on exogenous dietetic sources [4], and this may be the reason for this discrepancy. Concerning this point, since low extracellular levels of taurine trigger an increase in TauT cell expression and action* in vitro* [11, 12], having found unmodified plasma taurine levels between diabetic patients and controls could, at least partially, explain why taurine cell content was not significantly higher in diabetic individuals.

Plasma MDA, mirroring lipid peroxidation, was higher, even if not significantly, in patients with type 1 diabetes, and, in this case, it is not possible to speculate whether lipid peroxidation may contribute to modify TauT mRNA gene expression in MPCs. While no significant relationship was found between TauT mRNA gene expression and any single plasma marker of oxidative stress, TauT mRNA gene expression was inversely linked with plasma homocysteine. Interestingly this correlation was found only in the group of patients with diabetes, even if plasma levels of homocysteine were similar in patients with diabetes and in healthy controls, suggesting that plasma concentration of homocysteine could play a role in the regulation of TauT mRNA gene expression in diabetic subjects. In addition, even if the design of the study does not permit mechanistic conclusions, the inverse relationship between plasma homocysteine and TauT mRNA gene expression suggests that an increase in extracellular levels of homocysteine could be related to TauT mRNA downregulation. This is in agreement with the observation that homocysteine impairs active taurine transmembrane transport, as observed several years ago* in vitro* in synaptosomes, cultured neurons, and astrocytes [30]. More recently, just to reaffirm this finding in another cell model, it has been demonstrated that homocysteine inhibits taurine uptake* in vitro* from myocardial mitochondria of rats in a concentration-dependent manner [31].

Furthermore, TauT-mediated cell taurine influx might be modulated by intracellular synthesis of taurine originating from the transsulfuration pathway of homocysteine to cysteine and then to taurine [32], while, conversely, taurine supplementation might increase homocysteine metabolism, as happens in mice with cystathionine *β*-synthase-deficient homocystinuria [33].

In conclusion levels of homocysteine could be crucial in modifying cell TauT gene expression in patients with diabetes and differences in plasma levels of this amino acid as well as of taurine could determine differences in TauT gene expression as, for instance, noted between patients with type 1 and type 2 diabetes.

In agreement with what we have observed in type 2 diabetes [16], a further interesting point of this study is that type 1 diabetic patients without retinopathy, as compared to diabetic patients with retinopathy, are characterized by higher TauT mRNA gene expression in MPCs. TauT is involved in the active transport of taurine to the retinal pigment epithelial apical membrane [26] and a high concentration of taurine is essential for maintaining the structural and functional integrity of the retina [6]. This reduction in TauT mRNA gene expression in presence of retinopathy could thus represent an impairment in cell defense against chronic hyperglycemia, unanimously considered a main cause in the pathogenesis of diabetic retinopathy. Once again, however, what we have observed* “in vivo”* in diabetic patients appears to be different from TauT gene downregulation after the acute challenge of retinal epithelial and glial cells to high glucose levels* “in vitro”* [26, 34]. Our hypothesis is, however, reinforced by the fact that, interestingly, in animal models (diabetic rats), contrary to what happens in acute conditions, chronic exposure to high glucose levels seems to enhance active taurine uptake in retinal pigment epithelial cells, indirectly suggesting that diabetes is able to chronically stimulate* “in vivo”* TauT expression in these cells [35]. All this permits recalling that exposing the taurine-TauT system to higher glucose concentrations “*in vitro*” is completely different from testing it in a clinical condition such as diabetes, which is characterized by a much longer exposure time to extracellular high glucose levels and moreover includes a more complex biochemical milieu done by amino acids, lipids, or oxidative stress-associated metabolites.

Finally, no difference was found in TauT mRNA gene expression among patients with more advanced grades of retinopathy, probably due to the low number (*n* = 2) of patients with proliferative retinopathy.

## 5. Strengths and Limitations of the Study

A major strength of this study is represented by the further evidence that TauT gene expression is significantly upregulated in MPCs of patients with diabetes and is directly related to HbA1c, thus suggesting being a sort of response to the chronic impairment of plasma glucose control in diabetes. The study presents, however, some limitations: first, it is a cross-sectional study and does not permit evaluating any time-course of TauT mRNA expression and duration of disease, especially in relation to development of diabetes complications such as retinopathy. Secondly, the design of the study does not allow investigating whether TauT gene expression is modified at different target cell models.

## 6. Conclusions

In conclusion, this study highlights that TauT mRNA gene expression is upregulated in MPCs in type 1 diabetic patients, although to a much lesser extent than what we observed in type 2 diabetes [16]. We are not able to give explanation of the difference between the two types of diabetes: we can only exclude the effect of therapy or of a different metabolic control (which was similar to that observed in the study concerning type 2 diabetic patients). Probably, as highlighted above, a longer duration of diabetes, the different effect of plasma taurine concentrations (lower in the previous study concerning patients with type 2 diabetes), or a possible difference in plasma homocysteine levels might, at least partially, altogether explain such a difference. In addition, we can confirm that TauT mRNA expression in MPCs is positively related to metabolic control (HbA1c) and, finally, it seems to be inversely related to plasma homocysteine.

Even more interesting, however, is the fact that TauT mRNA expression is lower in patients with retinopathy, confirming what we observed in type 2 diabetes.

Finally, if our analyses excluded a significant relation between TauT mRNA gene expression and diabetes duration (*r* = −0.26; *P* = 0.06), further studies are needed to confirm the hypothesis that a way by which prolonged duration of hyperglycemia may induce the appearance of retinopathy can be also due to the progressive fading in upregulation of TauT gene expression.

## Figures and Tables

**Table 1:** Main characteristics of diabetic patients and control subjects.

	Controls	Type 1 diabetic patients	*P*
Number	33	35	
Age (yr)	41 ± 11	44 ± 12	NS
Sex (M/F)	12/21	18/17	NS
BMI (Kg/m^2^)	23.9 ± 3.3	25.2 ± 4.1	NS
Duration of diabetes (yr)	—	23.13 ± 9.44	—
Plasma glucose (mmol/L)	5.32 ± 0.48	8.29 ± 3.27	0.0001
HbA1c (%, mmol/mol)	5.38 ± 0.38, 34.64 ± 4.15	7.88 ± 1.07, 62.71 ± 11.70	0.0001
Plasma taurine (*µ*mol/L)	51.05 (19.30)	47.39 (23.46)	NS
Intracellular taurine (nmol/10^6^ cells)	38.33 (26.9)	54.19 (36.87)	NS
Plasma arginine (*µ*mol/L)	90.88 (19.49)	83.71 (23.41)	NS
Plasma homocysteine (*µ*mol/L)	11.4 (3.3)	11.1 (6.1)	NS
Asymmetric dimethylarginine (*µ*mol/L)	0.56 (0.22)	0.56 (0.13)	NS
Symmetric dimethylarginine (*µ*mol/L)	0.51 (0.28)	0.51 (0.14)	NS
Plasma malonyldialdehyde (*µ*mol/L)	3.55 (2.14)	4.47 (1.68)	NS
Intracellular carbonyls (nmol/mg)	1.37 (0.82)	1.30 (0.56)	NS
Lymphocytes (10^6^ cells/mL)	2.60 (0.86)	2.35 (0.79)	NS
Monocytes (10^6^ cells/mL)	0.50 (0.16)	0.45 (0.19)	NS
TauT mRNA gene expression (2^−ΔΔCt^)	1.00 (0.15)	1.32 (0.31)	0.0001
Urinary albumin/creatinine (*μ*g/mg)	5.277 (7.014)	5.601 (6.821)	NS

Data are shown as median (interquartile range) or mean ± SD.

**Table 2:** Correlation coefficients between TauT mRNA expression in MPCs of controls or type 1 diabetic patients and plasma glucose and markers of endothelial function or oxidative stress.

	Controls		Type 1 diabetic patients	
	*r*	*P*	*r*	*P*
Plasma glucose	0.27	NS	0.22	NS
Plasma taurine^*∗*^	0.25	NS	−0.29	NS
Cell taurine^*∗*^	0.24	NS	−0.03	NS
Plasma arginine^*∗*^	−0.11	NS	−0.23	NS
Plasma homocysteine^*∗*^	−0.14	NS	−0.39	0.02
Asymmetric dimethylarginine^*∗*^	−0.14	NS	0.31	NS
Symmetric dimethylarginine^*∗*^	−0.04	NS	0.30	NS
Plasma malonyldialdehyde^*∗*^	−0.03	NS	−0.23	NS
Cellular carbonyls^*∗*^	−0.03	NS	−0.06	NS

^*∗*^log transformed.

**Table 3:** TauT mRNA cell expression and plasma markers of oxidative stress or endothelial function in controls and in type 1 diabetic patients with or without retinopathy.

	No retinopathy	Retinopathy	*P*
Number	22	13	
Duration (yr)	17.50 ± 10.63	26.07 ± 9.37	0.02
HbA1c (%, mmol/mol)	7.77 ± 1.09, 61.50 ± 11.98	8.07 ± 1.04, 64.77 ± 11.38	NS
Plasma taurine (*µ*mol/L)	46.49 (23.79)	49.23 (18.58)	NS
Intracellular taurine (nmol/10^6^ cells)	63.19 (32.73)	43.34 (42.06)	NS
Plasma arginine (*µ*mol/L)	84.04 (16.72)	82.95 (44.71)	NS
Plasma homocysteine (*µ*mol/L)	10.90 (4.97)	13.1 (5.20)	NS
Asymmetric dimethylarginine (*µ*mol/L)	0.57 (0.13)	0.56 (0.10)	NS
Symmetric dimethylarginine (*µ*mol/L)	0.51 (0.15)	0.53 (0.22)	NS
Malonyldialdehyde (*µ*mol/L)	4.27 (1.71)	4.49 (1.83)	NS
Cellular carbonyls (nmol/mg)	1.22 (0.62)	1.50 (0.49)	NS
TauT mRNA gene expression (2^−ΔΔCt^)	1.36 (0.34)	1.16 (0.20)	0.02

Data are shown as median (interquartile range) or mean ± SD.
